# Post-Fracture Care After COVID-19 Mobility Restrictions in Older Adults with Femoral Neck Fracture: A Retrospective Pre–Post Study

**DOI:** 10.3390/healthcare14131858

**Published:** 2026-06-25

**Authors:** Ahmet Yılmaz, Mehmet Yiğit Gökmen, Osman Çiloğlu

**Affiliations:** 1 Department of Orthopaedics and Traumatology, Adana City Training and Research Hospital, University of Health Sciences, 01230 Adana, Türkiye; osmanciloglu@gmail.com; 2 Department of Orthopaedics and Traumatology, Faculty of Medicine, Çanakkale Onsekiz Mart University, 17110 Çanakkale, Türkiye; mehmet_yigit_gokmen@hotmail.com

**Keywords:** COVID-19, femoral neck fracture, hip fracture, older adults, mobility restriction, osteoporosis, rehabilitation, secondary fracture, postoperative care

## Abstract

**Background**: COVID-19-related mobility restrictions may have affected physical activity and post-fracture care in older adults. This study compared outcomes before and after age-based mobility restrictions, focusing on reported activity, documented combined postoperative physiotherapy and anti-osteoporotic pharmacologic treatment, and secondary fragility fractures. **Methods**: This retrospective single-center pre–post study included patients aged 65 years or older who underwent bipolar hemiarthroplasty for low-energy osteoporotic femoral neck fracture. Patients treated during the year before restrictions were compared with those treated during the post-restriction year. Outcomes included reported pre-fracture activity category, all-cause mortality, mobility among survivors, documented combined postoperative physiotherapy and anti-osteoporotic pharmacologic treatment, and secondary fragility fractures. **Results**: The pre-pandemic and post-restriction groups included 65 and 122 patients, respectively. Regular outdoor walking/activity before fracture was less frequent in the post-restriction group than in the pre-pandemic group (45.1% vs. 72.3%; *p* < 0.001), whereas home-limited activity was more frequent (54.9% vs. 27.7%). All-cause mortality during follow-up was 24.6% and 29.5%, respectively (*p* = 0.499). Mobility among survivors did not differ significantly (*p* = 0.832). Among survivors, documented combined postoperative physiotherapy and anti-osteoporotic pharmacologic treatment was uncommon: 14.3% and 17.4%, respectively (*p* = 0.809). Secondary fragility fractures were recorded only in the post-restriction group (8/86 survivors, 9.3%; *p* = 0.051). **Conclusions**: In this retrospective pre–post comparison, the post-restriction group showed a lower proportion of reported outdoor activity, while documented combined postoperative physiotherapy and anti-osteoporotic pharmacologic treatment remained uncommon among survivors. Secondary fragility fractures were observed only in the post-restriction group and should be interpreted with caution, given the exploratory design and limited number of events. Structured rehabilitation referral, osteoporosis treatment, fall-prevention strategies, and follow-up pathways remain important components of post-fracture care following femoral neck fracture.

## 1. Introduction

In December 2019, a cluster of pneumonia-like cases was reported in Wuhan, China, and the causative pathogen was subsequently identified as a novel coronavirus [1]. On 11 March 2020, the World Health Organization declared coronavirus disease 2019 (COVID-19) a global pandemic and called for urgent public health measures to limit transmission [2]. Many countries subsequently implemented social distancing, isolation, and stay-at-home restrictions. These measures were particularly strict for older adults because advanced age and comorbidity were associated with a higher risk of severe COVID-19-related outcomes. Although these restrictions were necessary for infection control, prolonged restrictions on outdoor mobility may have affected reported activity patterns, functional reserve, and access to routine post-fracture care in older adults.

Regular walking and low-intensity physical activity help preserve muscle strength, balance, postural control, bone health, and independence. Conversely, physical inactivity may accelerate deconditioning, sarcopenia, frailty progression, impaired gait stability, and dependence in daily activities [3]. Fear of infection, social isolation, reduced sunlight exposure, and limited access to outpatient rehabilitation may further increase musculoskeletal vulnerability during restriction periods. These changes are clinically important because falls and fragility fractures are multifactorial events influenced by bone quality, lower-extremity strength, balance impairment, comorbidities, medication use, cognitive or visual impairment, and environmental hazards [4].

Osteoporotic hip fractures remain one of the most serious consequences of low-energy falls in older adults. Hip fractures can also be considered a sentinel event reflecting frailty, sarcopenia, functional decline, osteoporosis, and increased future fracture risk. They are associated with substantial morbidity, mortality, loss of independence, and healthcare utilization. Physical activity, particularly regular walking, has been associated with a lower risk of falls and hip fractures, while structured, multicomponent exercise programs have been shown to improve frailty status and physical function in older adults [5,6,7]. Despite advances in surgical and perioperative care, mortality after fragility hip fracture remains clinically important, especially in older and frail populations [8]. In parallel, osteoporosis remains the underlying skeletal disease in many of these patients and requires systematic evaluation and treatment beyond surgical repair of the index fracture [9,10].

The COVID-19 pandemic created a unique healthcare context in which urgent hip fracture surgery often had to be preserved while routine outpatient care, rehabilitation access, osteoporosis evaluation, and secondary prevention pathways could be disrupted. Previous studies evaluating hip fractures during the COVID-19 pandemic have reported variable effects on fracture volume, patient characteristics, perioperative pathways, and outcomes across different healthcare settings and pandemic phases [11]. However, many studies have focused mainly on acute surgical pathways and perioperative outcomes. Less is known about differences in retrospectively reported pre-fracture activity categories and documented post-discharge care components, including physiotherapy, osteoporosis treatment, and secondary fragility fractures, after the onset of mobility restrictions. Prior fracture is one of the strongest predictors of future osteoporotic fracture [12].

Continuity of care after hip fracture is therefore essential. Current osteoporosis guidelines emphasize fracture risk assessment and timely initiation of appropriate pharmacologic treatment following a fragility fracture [9]. The osteoporosis treatment gap after hip fracture is a persistent healthcare problem and may become more difficult to address when outpatient visits, rehabilitation services, and follow-up pathways are disrupted. During public health crises, strategies such as telerehabilitation, structured home-based exercise, fall-prevention counseling, and Fracture Liaison Service models may help maintain post-fracture care when conventional outpatient services are limited [13,14].

The aim of this retrospective pre–post observational study was to describe and compare center-level femoral neck fracture case count, reported pre-fracture activity category, all-cause mortality during follow-up, postoperative mobility among survivors, documented combined postoperative physiotherapy and anti-osteoporotic pharmacologic treatment, and secondary fragility fracture occurrence among older adults treated with bipolar hemiarthroplasty for low-energy osteoporotic femoral neck fracture before and after the onset of COVID-19-related mobility restrictions.

## 2. Materials and Methods

### 2.1. Ethical Approval

This study was designed as a retrospective, single-center, pre–post observational cohort study. The analysis was exploratory and not designed to establish causal effects of COVID-19-related mobility restrictions. The study was conducted in the Department of Orthopedics and Traumatology of a tertiary training and research hospital. The study was approved by the local scientific ethics committee before data collection (approval date: 2 June 2021; approval number: 1425). The study was conducted in accordance with the principles of the Declaration of Helsinki. Because of the retrospective design and the use of anonymized clinical data, the ethics committee waived the requirement for informed consent. The study report was structured in accordance with the Strengthening the Reporting of Observational Studies in Epidemiology (STROBE) statement. A STROBE-style analytic cohort flow diagram was added to summarize group allocation, survival status, and the denominators used for outcome analyses based on the available retrospective data.

### 2.2. Study Periods and Participants

Public health measures in Türkiye included stay-at-home restrictions for adults aged 65 years or older beginning on 21 March 2020. In the first phase, older adults were fully restricted from leaving home for approximately 6 weeks, followed by a period of limited outdoor permission for 4–6 h per day. Thereafter, restrictions were intermittently modified, and routine outpatient access, rehabilitation services, and caregiver support may have varied during the study period. Therefore, the post-restriction period was considered a calendar-defined exposure period rather than a precisely measured individual-level restriction status.

For group definition, the pre-pandemic period was defined as the 1-year interval immediately before the onset of age-based mobility restrictions. Accordingly, patients treated between 21 March 2019 and 20 March 2020 were assigned to the pre-pandemic group (Group 1). The post-restriction period was defined as the 1-year interval after the onset of COVID-19-related mobility restrictions. Accordingly, patients treated between 21 March 2020 and 20 March 2021 were assigned to the post-restriction group (Group 2). Group allocation was based on the date of the index operation. Mortality and secondary fracture outcomes were assessed using the available follow-up after the index operation, with a minimum follow-up duration of 12 months in both groups. The maximum follow-up duration was 14 months in the pre-pandemic group and 16 months in the post-restriction group. Because follow-up in some pre-pandemic patients extended into the pandemic period, postoperative outcomes were interpreted according to the timing of the index fracture rather than the complete absence of later pandemic exposure.

### 2.3. Eligibility Criteria

Hospital digital records were reviewed to identify patients aged 65 years or older who underwent bipolar hemiarthroplasty for a low-energy osteoporotic femoral neck fracture during the study period. Eligible patients were 65 years or older and underwent bipolar hemiarthroplasty for a femoral neck fracture after a low-energy fall.

A low-energy osteoporotic femoral neck fracture was defined as a femoral neck fracture caused by a fall from standing height or lower in an older adult, without high-energy trauma, pathological fracture, or index periprosthetic fracture. Because bone mineral density measurements were not available for all patients, this definition was based on age, fracture mechanism, and clinical context rather than densitometric confirmation of osteoporosis in every patient. Bipolar hemiarthroplasty was performed for displaced femoral neck fractures according to the treating orthopedic surgeon’s assessment and the institutional treatment protocol for elderly patients with osteoporotic femoral neck fractures. No formal change in the institutional indication for bipolar hemiarthroplasty was implemented between the two study periods. All included patients had femoral neck fractures; therefore, trochanteric fractures were not included in the final analytic cohort. Detailed Garden classification and surgical variables could not be reliably reconstructed for all patients and were therefore not used as comparative variables.

The pre-pandemic group included 65 patients with a mean follow-up of 13 months (range, 12–14 months), and the post-restriction group included 122 patients with a mean follow-up of 13 months (range, 12–16 months). The total number of initially screened patients and detailed exclusion counts by reason could not be reliably reconstructed from the retrospective institutional records; therefore, the analytic flow diagram summarizes the final analytic cohort, group allocation, survival status, and outcome-specific denominators.

The following exclusion criteria were applied before final analysis: high-energy trauma; pathological fracture due to malignancy; index periprosthetic fracture; trochanteric or subtrochanteric fracture; pre-fracture non-ambulatory status; additional fractures at the time of the index femoral neck fracture; active COVID-19 infection or a positive COVID-19 test during the index admission; prosthetic infection or dislocation requiring additional major treatment during follow-up; loss to follow-up; and inability to obtain reliable follow-up information from either the patient or a first-degree relative/caregiver. Active COVID-19 infection was defined as a documented positive SARS-CoV-2 test or a clinical diagnosis of COVID-19 during the index admission. Patients with severe cognitive impairment were excluded only when reliable information on pre-fracture activity, postoperative mobility, mortality, or secondary fracture occurrence could not be obtained from a caregiver.

### 2.4. Data Collection and Variables

Clinical data were obtained retrospectively from the hospital’s digital archive. Demographic data, fracture characteristics, operative treatment records, comorbidities, discharge notes, outpatient follow-up records, mortality information, rehabilitation documentation, anti-osteoporotic treatment records, and secondary fracture events were reviewed. When routine follow-up information was incomplete, missing outcome data were supplemented by telephone contact with patients or relatives. Telephone follow-up was performed by members of the study team using the same structured question set for both study periods. The structured question set focused on survival status, current mobility level, receipt of postoperative physiotherapy, use of anti-osteoporotic medication, and occurrence of any new low-energy fracture after the index femoral neck fracture. When possible, patient or caregiver reports were cross-checked with hospital records, outpatient notes, imaging reports, or treatment documentation.

The variables recorded were age, sex, follow-up duration, comorbidities, pre-fracture mobility status, reported pre-fracture activity category, all-cause mortality during follow-up, postoperative mobility among survivors, documented combined postoperative physiotherapy and anti-osteoporotic pharmacologic treatment, and secondary fragility fractures during follow-up. Because separate documentation of postoperative physiotherapy and anti-osteoporotic pharmacologic treatment was incomplete in a subset of patients, these components were not analyzed as separate endpoints. The combined endpoint was defined as documented receipt of both postoperative physiotherapy and anti-osteoporotic pharmacologic treatment.

Comorbidity status was recorded as the presence or absence of at least one documented chronic systemic disease, such as hypertension, diabetes mellitus, coronary artery disease, chronic pulmonary disease, chronic kidney disease, cerebrovascular disease, or neurologic disease affecting mobility. Pre-fracture mobility status was categorized as independent or assisted ambulation based on the need for walking support before the index fracture.

Pre-fracture activity was categorized as regular outdoor walking/activity or home-limited activity based on patient or caregiver report. Regular outdoor walking/activity was defined as light-to-moderate walking or outdoor activity approximately 3–5 days per week for 30–60 min before fracture. Home-limited activity was defined as predominantly indoor mobilization or restricted household ambulation. Pre-fracture activity referred to the usual activity pattern in the period immediately before the index fracture. This variable was not based on a validated physical activity questionnaire or objective activity monitoring.

Postoperative mobility among survivors was categorized as independent ambulation, assisted ambulation, or immobility. Postoperative mobility was assessed at the final available follow-up among surviving patients only. Independent ambulation was defined as walking without regular human assistance, assisted ambulation as walking with a cane, walker, or caregiver support, and immobility as being unable to ambulate functionally despite assistance. This categorical assessment did not capture walking distance, gait speed, endurance, fear of falling, balance confidence, or participation level.

Postoperative physiotherapy was defined as documented inpatient, outpatient, or home-based physiotherapy after discharge. Anti-osteoporotic treatment was defined as documented pharmacologic therapy for osteoporosis prescribed after the index fracture, including bisphosphonates, denosumab, teriparatide, or other anti-resorptive or anabolic medications. Calcium or vitamin D supplementation alone was not considered sufficient to define anti-osteoporotic pharmacologic treatment. Treatment adherence, persistence, duration, intensity, and adequacy of long-term osteoporosis care were not consistently available in the retrospective records.

### 2.5. Outcome Measures

The main outcomes of interest were pre–post differences in the documented post-fracture care profile of older adults treated surgically for osteoporotic femoral neck fractures, with particular emphasis on reported pre-fracture activity category, documented combined postoperative physiotherapy and anti-osteoporotic pharmacologic treatment, and the occurrence of secondary fragility fractures.

Additional outcomes included postoperative all-cause mortality and postoperative mobility status among survivors at final follow-up. Postoperative mortality was defined as all-cause death recorded during the available postoperative follow-up, with a minimum follow-up duration of 12 months. The observed number of eligible patients with femoral neck fractures in each period was evaluated only as a descriptive, center-level case-count indicator and was not interpreted as a population-level incidence measure.

Secondary fragility fractures were defined as new low-energy osteoporotic fractures occurring during follow-up after the index femoral neck fracture operation. Postoperative mobility and secondary fracture occurrence were analyzed among survivors because these outcomes required follow-up assessment after the index operation.

### 2.6. Statistical Analysis

Continuous variables were summarized as means with ranges. Because age and follow-up duration were reported descriptively, no inferential comparisons were performed on continuous variables. Categorical variables are presented as counts and percentages. Between-group comparisons of categorical variables were performed using the chi-square test or Fisher’s exact test, as appropriate. Fisher’s exact test was preferred when expected cell counts were small. For selected binary outcomes, 95% confidence intervals for group-specific proportions were reported when appropriate.

Because the total older-adult population at risk, total emergency admissions, all orthopedic trauma volumes, and referral-flow denominators were not available, no inferential test was applied to the difference in the observed number of eligible patients with femoral neck fractures between periods; this comparison is reported descriptively. Therefore, the difference in the observed number of eligible patients should not be interpreted as a true change in the population-level incidence of femoral neck fractures.

The available dataset did not support robust multivariable adjustment because several clinically relevant confounders, including frailty, cognitive status, ASA status, previous fragility fracture, prior osteoporosis treatment, discharge destination, and treatment adherence, were not consistently available. Therefore, age- and sex-only adjustment was not performed. No imputation was performed for missing data. Patients with incomplete core outcome information that could not be resolved through hospital records or telephone follow-up were excluded from the final analysis. All analyses were two-sided, and a *p*-value < 0.05 was considered statistically significant. Given the small number of secondary fracture events, the secondary-fracture analysis was considered exploratory. Statistical analyses were performed using IBM SPSS Statistics, version 26 (IBM Corp., Armonk, NY, USA).

## 3. Results

### 3.1. Study Cohort and Included Patients

**Figure 1:**
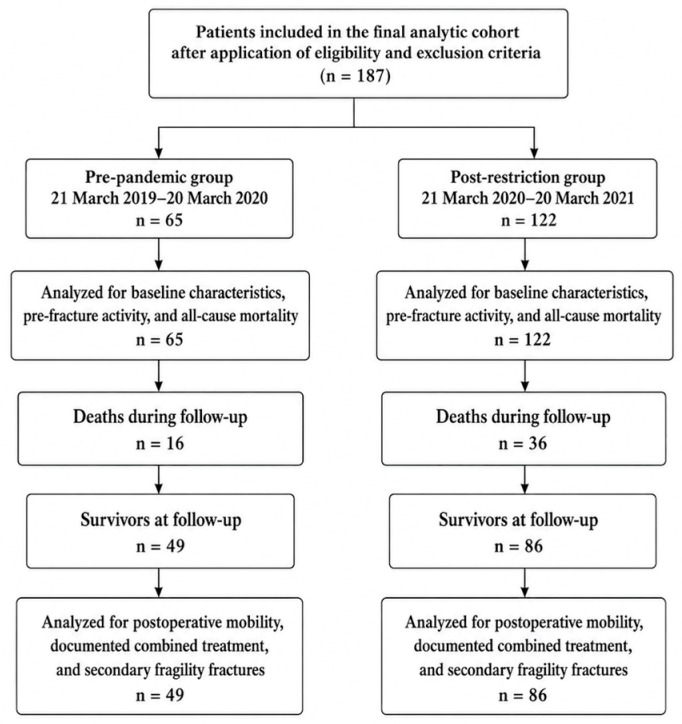
Analytic cohort flow diagram. The final analytic cohort was divided into pre-pandemic and post-restriction groups. Mortality was analyzed in all included patients, whereas postoperative mobility, documented combined postoperative physiotherapy and anti-osteoporotic pharmacologic treatment, and secondary fragility fractures were analyzed among survivors.

The final analytic cohort included 187 patients after application of the available eligibility and exclusion criteria. The pre-pandemic group included 65 patients, and the post-restriction group included 122 patients. The mean follow-up duration was 13 months in both groups. The analytic cohort flow, including group allocation, survival status, and outcome-specific denominators, is shown in Figure 1. Study periods, cohort size, follow-up duration, and baseline demographic and clinical characteristics are presented in Table 1.

### 3.2. Baseline Demographic and Clinical Characteristics

The mean age was 76.7 years in the pre-pandemic group and 73.7 years in the post-restriction group. Female sex was recorded in 46 patients in the pre-pandemic group and 84 patients in the post-restriction group, with no statistically significant between-group difference (*p* = 0.868).

At least one comorbidity was present in 58 patients in the pre-pandemic group and 114 patients in the post-restriction group (*p* = 0.397). Pre-fracture independent ambulation was documented in 53 patients in the pre-pandemic group and 96 patients in the post-restriction group (*p* = 0.706). Baseline demographic and clinical characteristics are summarized in Table 1.

### 3.3. Reported Pre-Fracture Activity Category

Regular outdoor walking or other outdoor activities before fracture were reported by 47 patients in the pre-pandemic group and 55 in the post-restriction group. Home-limited activity was reported in 18 and 67 patients, respectively.

The distribution of reported pre-fracture activity categories differed between groups (*p* < 0.001). Reported pre-fracture activity data are presented in Table 2.

### 3.4. All-Cause Mortality During Follow-Up and Mobility Among Survivors

All-cause mortality during follow-up occurred in 16 of 65 patients in the pre-pandemic group and 36 of 122 patients in the post-restriction group. The difference in mortality between groups was not statistically significant (*p* = 0.499).

Among survivors, independent ambulation was recorded in 15 of 49 patients in the pre-pandemic group and 24 of 86 patients in the post-restriction group. Assisted ambulation was recorded in 23 and 45 survivors, respectively. Immobility was recorded in 11 and 17 survivors, respectively. The distribution of postoperative mobility categories among survivors did not differ significantly between groups (*p* = 0.832). All-cause mortality during follow-up and mobility outcomes among survivors are shown in Table 3.

### 3.5. Documented Combined Postoperative Physiotherapy and Anti-Osteoporotic Pharmacologic Treatment, and Secondary Fragility Fractures

Documented combined postoperative physiotherapy and anti-osteoporotic pharmacologic treatment was recorded in 7 of 49 survivors in the pre-pandemic group and 15 of 86 survivors in the post-restriction group. The difference between groups was not statistically significant (*p* = 0.809).

No secondary fragility fracture was recorded among survivors in the pre-pandemic group, whereas secondary fragility fractures were recorded in 8 of 86 survivors in the post-restriction group (*p* = 0.051). Documented combined postoperative physiotherapy and anti-osteoporotic pharmacologic treatment and secondary fragility fracture outcomes are summarized in Table 4.

The secondary fragility fractures recorded in the post-restriction group consisted of distal radius fractures in three patients, contralateral hip fractures in two patients, a proximal humerus fracture in one patient, a vertebral compression fracture in one patient, and a low-energy periprosthetic fracture in one patient.

## 4. Discussion

This retrospective pre–post study evaluated older adults who underwent bipolar hemiarthroplasty for low-energy osteoporotic femoral neck fracture before and after COVID-19-related mobility restrictions. The principal findings were as follows: the observed center-level number of included surgically treated femoral neck fracture patients was higher in the post-restriction period; reported regular outdoor walking/activity before fracture was less frequent, whereas home-limited activity was more common; all-cause mortality during follow-up and categorical mobility status among survivors did not differ significantly between periods; documented combined postoperative physiotherapy and anti-osteoporotic pharmacologic treatment was uncommon in both groups; and secondary fragility fractures were recorded only in the post-restriction group. These findings should be interpreted as descriptive associations from a single-center pre–post cohort and not as evidence that mobility restrictions directly caused the observed outcomes.

The higher number of eligible patients with femoral neck fractures during the post-restriction period should be interpreted with caution. Because regional population denominators, total emergency department admissions, overall orthopedic trauma volume, and referral-flow data were not available, this finding cannot be used to infer a true population-level increase in femoral neck fracture incidence. Instead, it should be regarded as a center-level observation reflecting the number of patients with surgically treated femoral neck fractures included in the study institution during the post-restriction period. Previous studies have reported heterogeneous changes in hip fracture volume and outcomes during the COVID-19 pandemic [11,15,16]. Tanaka et al. reported changes in hip fracture patterns among older patients before and after the pandemic in a multicenter Japanese cohort [11], while other studies demonstrated that hip fracture demographics, perioperative pathways, and outcomes varied across different pandemic phases and healthcare settings [15,16]. This variability suggests that center-level fracture case numbers may be influenced by multiple local factors, including mobility policies, household structure, referral pathways, hospital capacity, and access to urgent orthopedic services. Therefore, the difference in the observed number of included patients should be interpreted as institution-level data rather than epidemiological incidence evidence.

The main between-group difference observed in this study was the distribution of reported pre-fracture activity categories. Regular outdoor walking/activity was less frequent in the post-restriction group, while home-limited activity was more common. Osteoporosis is characterized by reduced bone mass and microarchitectural deterioration, which predispose older adults to low-energy fractures [17,18]. Physical activity and walking have long been associated with reduced risk of falls and hip fractures in older adults [6,19,20]. Walking may help preserve lower-extremity strength, balance, postural control, and independence, whereas prolonged physical inactivity may accelerate sarcopenia, frailty, and functional decline. Recent evidence also supports the importance of structured and multicomponent exercise interventions in older adults, particularly for improving frailty-related parameters such as muscle strength, gait speed, and balance [21]. However, the activity variable in the present study was based on retrospective patient or caregiver report and was not measured with a validated physical activity scale. Therefore, this finding should be interpreted as a difference in reported pre-fracture activity category rather than as an objective measurement of physical activity decline.

Postoperative mortality did not differ significantly between the two periods. This finding is consistent with several reports suggesting that urgent hip fracture surgery was generally maintained during the pandemic despite major pressure on healthcare systems [11,15,16]. In the Japanese multicenter study by Tanaka et al., waiting time for surgery, length of hospital stay, and in-hospital mortality did not differ significantly across the pre-COVID, early-COVID, and late-COVID periods [11]. Similarly, Nazemi et al. reported that hip fracture care continued during the pandemic, although changes in demographics and length of stay were observed [16]. The present study differs from many pandemic hip fracture studies by excluding patients with active COVID-19 infection or positive COVID-19 testing during the index admission. This may partly explain why mortality did not differ significantly between periods. Other possible explanations include preservation of urgent surgical pathways, limited sample size, and the use of broad follow-up mortality rather than more granular 30-day, 90-day, or 1-year time-point analyses.

The absence of a significant difference in broad postoperative mobility categories should also be interpreted in context. Although the proportions of independent ambulation, assisted ambulation, and immobility among survivors were similar between groups, categorical mobility status may not fully capture differences in walking endurance, confidence, balance, fear of falling, or participation in daily life. Hip fracture recovery is not determined solely by surgical survival or the ability to ambulate at final follow-up; it also depends on the quality, continuity, and intensity of post-discharge rehabilitation. The low rate of documented combined postoperative physiotherapy and anti-osteoporotic pharmacologic treatment across both periods suggests that documentation of both post-discharge care components was uncommon in this cohort. COVID-19 substantially affected the delivery of physiotherapy, and telerehabilitation emerged as a potential alternative model to preserve continuity of musculoskeletal rehabilitation when face-to-face care is restricted [13]. From a healthcare-systems perspective, these findings support consideration of structured discharge pathways that include rehabilitation referral, home-based exercise instructions, fall-prevention counseling, and mechanisms for follow-up when conventional outpatient visits are disrupted.

Secondary fragility fractures were recorded in 8 of 86 survivors in the post-restriction group and in none in the pre-pandemic group. Prior fracture is one of the strongest predictors of subsequent fragility fracture, and previous studies have shown that the risk of a second hip fracture or another osteoporotic fracture remains substantial after an initial fragility fracture [12,22,23]. The anatomical distribution of secondary fractures in the present study, including distal radius, contralateral hip, proximal humerus, vertebral, and periprosthetic fractures, is consistent with the broader spectrum of major osteoporotic fractures. Nevertheless, the small number of events, a survivor-based analysis, unequal maximum follow-up durations, incomplete information on osteoporosis treatment and fall risk, and the absence of competing-risk modeling limit the interpretation of this finding. Therefore, secondary fracture occurrence should be regarded as an exploratory observation rather than confirmatory evidence of an effect of the post-restriction period.

The low rate of documented combined postoperative physiotherapy and anti-osteoporotic pharmacologic treatment was an important post-discharge observation. International guidelines emphasize that patients with fragility fractures should undergo fracture risk assessment and receive appropriate osteoporosis treatment to reduce the risk of subsequent fractures [9]. Recent population-level data continue to show substantial underuse of osteoporosis medication after hip fracture [24]. Current reviews also emphasize that osteoporosis remains a major contributor to fracture burden in older adults and that appropriate pharmacologic management is central to secondary prevention [10]. Similarly, a recent tertiary-center study in Türkiye reported a large post-fracture osteoporosis treatment gap, highlighting that this problem is also relevant in the national healthcare context [25]. However, the present study evaluated documentation of both treatment components rather than actual treatment uptake, adherence, persistence, treatment duration, bone mineral density assessment, or the adequacy of long-term osteoporosis care. Therefore, these findings should be interpreted as limited documentation of receipt of post-fracture treatment, not as a comprehensive assessment of care continuity.

From a clinical perspective, the low documented receipt of combined postoperative physiotherapy and anti-osteoporotic pharmacologic treatment highlights the need to consider structured post-fracture pathways after hip fracture. Fracture Liaison Service models may provide a practical framework for identifying patients after a fragility fracture, evaluating osteoporosis and fall risk, initiating appropriate treatment, and monitoring adherence over time [14]. During public health crises, early rehabilitation referral, home-based exercise instructions, fall-prevention counseling, caregiver education, initiation of osteoporosis treatment, and telephone or remote follow-up may help support post-discharge care when routine outpatient access is limited [13,14].

Digital health and telemedicine models may also support medication checks, rehabilitation follow-up, and fall-prevention counseling, although inequitable access and limited technological literacy remain important barriers [26]. These strategies were not directly evaluated in the present study; therefore, they should be considered practical implications for future care models rather than conclusions derived from the study data.

This study has several limitations. First, the retrospective single-center design limits generalizability. Second, the exclusion of patients with incomplete or unreliable follow-up information may have introduced selection bias and affected the estimated proportions of mortality, postoperative mobility, documented combined treatment, and secondary fracture occurrence. Because this study was based on retrospective institutional records, the total number of initially screened patients and detailed exclusion counts by reason could not be reliably reconstructed. Third, the reported pre-fracture activity category was based on patient or caregiver report and was not measured using a validated physical activity questionnaire or objective activity monitoring. Fourth, the study did not include bone mineral density measurements, ASA status, cognitive status, frailty scores, fall-risk scores, standardized functional outcome measures, nutritional status, time to surgery, discharge destination, detailed medication history, treatment duration, treatment intensity, adherence data, or socioeconomic variables. Fifth, the observed increase in eligible patients with femoral neck fractures cannot be interpreted as a true increase in incidence because population-level and referral-flow denominators were unavailable. Sixth, the analysis was not designed to isolate the separate effects of fear of infection, social isolation, reduced outpatient access, delayed rehabilitation, altered family support, or healthcare-resource reallocation. Finally, multivariable modeling was not performed because of the incomplete availability of key confounders and the limited number of events, particularly for secondary fragility fractures. These limitations restrict causal interpretation and support the view that the findings are exploratory.

Despite these limitations, the study has several strengths. It focuses on a vulnerable older population treated surgically for low-energy osteoporotic femoral neck fracture, compares two clearly defined public health periods, includes a comparable minimum follow-up duration, and evaluates both acute outcomes and selected post-discharge care-related outcomes. By excluding patients with active COVID-19 infection during the index admission, the study specifically describes outcomes across public health periods without directly mixing these findings with the perioperative effects of SARS-CoV-2 infection.

## 5. Conclusions

In this retrospective single-center pre–post study of older adults treated surgically for low-energy osteoporotic femoral neck fracture, the post-restriction group had a lower proportion of reported regular outdoor walking/activity and a higher proportion of home-limited activity before fracture. All-cause mortality during follow-up and broad mobility categories among survivors did not differ significantly between the two periods. Documented combined postoperative physiotherapy and anti-osteoporotic pharmacologic treatment remained uncommon in both groups. Secondary fragility fractures were recorded only in the post-restriction group, but the small number of events and exploratory nature of this survivor-based analysis limit interpretation. Future prospective multicenter studies with standardized functional assessment, fracture risk evaluation, rehabilitation data, osteoporosis treatment adherence, and longer follow-up are needed. Structured rehabilitation referral, osteoporosis treatment, fall-prevention counseling, patient and caregiver education, and follow-up mechanisms should be considered as important components of post-fracture care during and after public health crises.

## Figures and Tables

**Table 1:** Study cohort and baseline characteristics.

Variable	Pre-Pandemic Group (n = 65)	Post-Restriction Group (n = 122)	*p*-Value/Interpretation
**Study period**	21 March 2019–20 March 2020	21 March 2020–20 March 2021	Pre–post comparison
**Patients in final analytic cohort**	65	122	Descriptive; no inferential test
**Mean follow-up, months, mean (range)**	13 (12–14)	13 (12–16)	Descriptive
**Age, years, mean (range)**	76.7 (65–94)	73.7 (65–98)	Descriptive
**Female sex**	46 (70.8%)	84 (68.9%)	0.868
**At least one comorbidity**	58 (89.2%)	114 (93.4%)	0.397
**Pre-fracture independent ambulation**	53 (81.5%)	96 (78.7%)	0.706
**Pre-fracture assisted ambulation**	12 (18.5%)	26 (21.3%)	—

Data are presented as n (%) unless otherwise indicated. All included patients had femoral neck fractures. The number of patients in the final analytic cohort was reported descriptively because population-level and referral-flow denominators were unavailable; therefore, no inferential test was performed for this row. *p*-values were calculated using two-sided Fisher’s exact test for categorical comparisons.

**Table 2:** Reported pre-fracture activity category.

Activity Category	Pre-Pandemic Group (n = 65)	Post-Restriction Group (n = 122)	*p*-Value
**Regular outdoor walking/activity**	47 (72.3%)	55 (45.1%)	<0.001
**Home-limited activity**	18 (27.7%)	67 (54.9%)	—

The *p*-value compares the distribution of reported regular outdoor walking/activity versus home-limited activity between groups. Regular outdoor walking/activity was defined as light-to-moderate activity for approximately 3–5 days per week, lasting 30–60 min, in the period immediately before the index fracture.

**Table 3:** All-cause mortality during follow-up and mobility among survivors.

Outcome	Pre-Pandemic Group	Post-Restriction Group	*p*-Value
**All-cause mortality during follow-up**	16/65 (24.6%; 95% CI, 15.8–36.3)	36/122 (29.5%; 95% CI, 22.1–38.1)	0.499
**Survivors at follow-up**	49/65 (75.4%)	86/122 (70.5%)	—
**Independent ambulation among survivors**	15/49 (30.6%)	24/86 (27.9%)	0.832 *
**Assisted ambulation among survivors**	23/49 (46.9%)	45/86 (52.3%)	—
**Immobile among survivors**	11/49 (22.4%)	17/86 (19.8%)	—

Data are presented as n/N (%) unless otherwise indicated. Confidence intervals were calculated for the mortality proportions. * *p*-value for the three-category postoperative mobility distribution among survivors. Mortality was compared using a two-sided Fisher’s exact test.

**Table 4:** Documented combined postoperative physiotherapy and anti-osteoporotic pharmacologic treatment, and secondary fragility fracture outcomes among survivors.

Outcome	Pre-Pandemic Group (n = 49 Survivors)	Post-Restriction Group (n = 86 Survivors)	*p*-Value
**Documented combined postoperative physiotherapy and anti-osteoporotic pharmacologic treatment**	7 (14.3%; 95% CI, 7.1–26.7)	15 (17.4%; 95% CI, 10.9–26.8)	0.809
**Secondary fragility fracture during follow-up**	0 (0.0%; 95% CI, 0.0–7.3)	8 (9.3%; 95% CI, 4.8–17.3)	0.051

Data are presented as n (%). *p*-values were calculated using two-sided Fisher’s exact test. Documented combined postoperative physiotherapy and anti-osteoporotic pharmacologic treatment and secondary fragility fracture occurrence were assessed among survivors. Confidence intervals were calculated for selected binary outcomes.

## Data Availability

The data are not publicly available because of patient privacy and institutional restrictions. De-identified data may be made available from the corresponding author upon reasonable request and with appropriate institutional permission.

## References

[1] Huang C., Wang Y., Li X., Ren L., Zhao J., Hu Y., Zhang L., Fan G., Xu J., Gu X. (2020). Clinical Features of Patients Infected with 2019 Novel Coronavirus in Wuhan, China. Lancet.

[2] Cucinotta D., Vanelli M. (2020). WHO Declares COVID-19 a Pandemic. Acta Biomed..

[3] Said C.M., Batchelor F., Duque G. (2020). Physical Activity and Exercise for Older People During and After the Coronavirus Disease 2019 Pandemic: A Path to Recovery. J. Am. Med. Dir. Assoc..

[4] Mazo G.Z., Fank F., Franco P.S., Capanema B.D.S.V., Pereira F.D.S. (2022). Impact of Social Isolation on Physical Activity and Factors Associated with Sedentary Behavior in Older Adults During the COVID-19 Pandemic. J. Aging Phys. Act..

[5] Ambrose A.F., Cruz L., Paul G. (2015). Falls and Fractures: A Systematic Approach to Screening and Prevention. Maturitas.

[6] Feskanich D., Willett W., Colditz G. (2002). Walking and Leisure-Time Activity and Risk of Hip Fracture in Postmenopausal Women. JAMA.

[7] Yang X., Li S., Xu L., Liu H., Li Y., Song X., Bao J., Liao S., Xi Y., Guo G. (2024). Effects of Multicomponent Exercise on Frailty Status and Physical Function in Frail Older Adults: A Meta-Analysis and Systematic Review. Exp. Gerontol..

[8] Daraphongsataporn N., Saloa S., Sriruanthong K., Philawuth N., Waiwattana K., Chonyuen P., Pimolbutr K., Sucharitpongpan W. (2020). One-Year Mortality Rate after Fragility Hip Fractures and Associated Risk in Nan, Thailand. Osteoporos. Sarcopenia.

[9] Gregson C.L., Armstrong D.J., Avgerinou C., Bowden J., Cooper C., Douglas L., Edwards J., Gittoes N.J.L., Harvey N.C., Kanis J.A. (2025). The 2024 UK Clinical Guideline for the Prevention and Treatment of Osteoporosis. Arch. Osteoporos..

[10] Ye C., Ebeling P., Kline G. (2025). Osteoporosis. Lancet.

[11] Tanaka S., Osawa Y., Takegami Y., Okui N., Yamauchi K.-I., Aoki Y., Imagama S. (2024). Changes of Hip Fracture in Older Patients before and after the COVID-19 Pandemic: A Retrospective Multicentre Study in Japan. BMC Musculoskelet. Disord..

[12] Klotzbuecher C.M., Ross P.D., Landsman P.B., Abbott T.A., Berger M. (2000). Patients with Prior Fractures Have an Increased Risk of Future Fractures: A Summary of the Literature and Statistical Synthesis. J. Bone Miner. Res..

[13] Dierick F., Pierre A., Profeta L., Telliez F., Buisseret F. (2021). Perceived Usefulness of Telerehabilitation of Musculoskeletal Disorders: A Belgium-France Pilot Study during Second Wave of COVID-19 Pandemic. Healthcare.

[14] Kocijan R., Haschka J., Kraus D.A., Pfender A., Frank S., Zwerina J., Behanova M. (2024). Perspectives on Fracture Liaison Service in Austria: Clinical and Economic Considerations. Front. Endocrinol..

[15] Case T., Kricfalusi M., Ruckle D., Razzouk J., Dahan A., Elsissy J.G., Schneiderman B.A. (2024). Evolving Effects of the COVID-19 Pandemic on Hip Fracture Outcomes: A Retrospective Comparison of Pre, Early, and Late Pandemic Timepoints. J. Am. Acad. Orthop. Surg. Glob. Res. Rev..

[16] Nazemi A.K., Al-Humadi S.M., Tantone R., Hays T.R., Bowen S.N., Komatsu D.E., Divaris N. (2021). Hip Fractures Before and During the COVID-19 Pandemic: Comparative Demographics and Outcomes. Geriatr. Orthop. Surg. Rehabil..

[17] (1991). Consensus Development Conference: Prophylaxis and Treatment of Osteoporosis. Am. J. Med..

[18] Colón-Emeric C.S., Saag K.G. (2006). Osteoporotic Fractures in Older Adults. Best Pract. Res. Clin. Rheumatol..

[19] Gregg E.W., Pereira M.A., Caspersen C.J. (2000). Physical Activity, Falls, and Fractures among Older Adults: A Review of the Epidemiologic Evidence. J. Am. Geriatr. Soc..

[20] Yamazaki S., Ichimura S., Iwamoto J., Takeda T., Toyama Y. (2004). Effect of Walking Exercise on Bone Metabolism in Postmenopausal Women with Osteopenia/Osteoporosis. J. Bone Miner. Metab..

[21] Martínez-Montas G.F., Sanz-Matesanz M., Benítez-Sillero J.D.D., Martínez-Aranda L.M. (2025). Prevention and Mitigation of Frailty Syndrome in Institutionalised Older Adults Through Physical Activity: A Systematic Review. Healthcare.

[22] Scaglione M., Fabbri L., Di Rollo F., Bianchi M.G., Dell’omo D., Guido G. (2013). The Second Hip Fracture in Osteoporotic Patients: Not Only an Orthopaedic Matter. Clin. Cases Miner. Bone Metab..

[23] Nymark T., Lauritsen J.M., Ovesen O., Röck N.D., Jeune B. (2006). Short Time-Frame from First to Second Hip Fracture in the Funen County Hip Fracture Study. Osteoporos. Int..

[24] Llopis-Cardona F., Rodríguez-Bernal C.L., Hurtado I., Espallargues M., García N., Gorostiza I., Gorricho J., Librero J., Millán E., Modroño G. (2025). Contemporary Pharmacological Management of Patients after Hip Fracture: A Population-Based Cohort of over 120,000 Patients in Spain. Osteoporos. Int..

[25] Çakır S.D., Yerli M., Sonmez M., Adas M. (2025). Osteoporosis Treatment Gap after Hip Fracture: Data from a Tertiary Center in Turkey. Arch. Osteoporos..

[26] Anastasiadou O., Tsipouras M., Mpogiatzidis P., Angelidis P. (2025). Digital Healthcare Innovative Services in Times of Crisis: A Literature Review. Healthcare.

